# Critically ill patients with community-onset intraabdominal infections: Influence of healthcare exposure on resistance rates and mortality

**DOI:** 10.1371/journal.pone.0223092

**Published:** 2019-09-26

**Authors:** Emilio Maseda, Sofía Ramírez, Pedro Picatto, Eva Peláez-Peláez, Carlos García-Bernedo, Nazario Ojeda-Betancur, Gerardo Aguilar, Beatriz Forés, Jorge Solera-Marín, María Aliaño-Piña, Eduardo Tamayo, Fernando Ramasco, Raquel García-Álvarez, Ada González-Lisorge, María-José Giménez, Alejandro Suárez-de-la-Rica, for the HELP Investigators and the Perioperative Infection Research Group

**Affiliations:** 1 Anesthesiology and Surgical Critical Care Dpt., Hospital Universitario La Paz, Madrid, Spain; 2 Universidad Autónoma de Madrid, Madrid, Spain; 3 Anesthesiology and Surgical Critical Care Dpt., Hospital Universitario Central de Asturias, Oviedo, Spain; 4 Anesthesiology and Surgical Critical Care Dpt., Hospital Universitario Vall d’Hebron, Barcelona, Spain; 5 Anesthesiology and Surgical Critical Care Dpt., Hospital del Mar, Barcelona, Spain; 6 Anesthesiology and Surgical Critical Care Dpt., Hospital Universitario de Gran Canaria Dr. Negrín, Las Palmas de Gran Canaria, Spain; 7 Anesthesiology and Surgical Critical Care Dpt., Hospital Clínico Universitario, Valencia, Spain; 8 Anesthesiology and Surgical Critical Care Dpt., Hospital Universitario Basurto, Bilbao, Spain; 9 Anesthesiology and Surgical Critical Care Dpt., Hospital Universitario de Canarias, Santa Cruz de Tenerife, Spain; 10 Anesthesiology and Surgical Critical Care Dpt., Hospital Virgen de la Salud, Toledo, Spain; 11 Anesthesiology and Surgical Critical Care Dpt., Hospital Clínico Universitario, Valladolid, Spain; 12 Anesthesiology and Surgical Critical Care Dpt., Hospital Universitario de la Princesa, Madrid, Spain; 13 Anesthesiology and Surgical Critical Care Dpt., Hospital Universitario 12 de Octubre, Madrid, Spain; 14 Anesthesiology and Surgical Critical Care Dpt., Hospital Clínico Universitario Virgen de la Arrixaca, Murcia, Spain; 15 PRISM-AG, Madrid, Spain; 16 Universidad Europea, Madrid, Spain; University of Notre Dame Australia, AUSTRALIA

## Abstract

The concept of healthcare-associated infections (as opposed to hospital-acquired infections) in intraabdominal infections (IAIs) is scarcely supported by data in the literature. The aim of the present study was to analyse community-onset IAIs (non-postoperative/non-nosocomial) in patients admitted to intensive care units (ICUs), to investigate differences in resistance patterns linked to healthcare exposure and mortality-associated factors. A one-year prospective observational study (17 Spanish ICUs) was performed distributing cases as healthcare-associated infections (HCAI), community-acquired infections (CAI) and immunocompromised patients (ICP). Bacteria producing extended-spectrum β-lactamases (ESBL) and/or carbapenemase (CPE), high-level aminoglycoside- and/or methicillin- and/or vancomycin- resistance were considered antimicrobial resistant (AMR). Mortality-associated factors were identified by regression multivariate analysis. Of 345 patients included (18.8% HCAI, 6.1% ICP, 75.1% CAI), 51.6% presented generalized peritonitis; 32.5% were >75 years (55.4% among HCAI). Overall, 11.0% cases presented AMR (7.0% ESBL- and/or CPE), being significantly higher in HCAI (35.4%) vs. CAI (5.8%) (p<0.001) vs. ICP (0%) (p = 0.003). Overall 30-day mortality was 14.5%: 23.1% for HCAI and 11.6% for CAI (p = 0.016). Mortality (R^2^ = 0.262, p = 0.021) was positively associated with age >75 years (OR = 6.67, 95%CI = 2.56–17.36,p<0.001), *Candida* isolation (OR = 3.05, 95%CI = 1.18–7.87,p = 0.022), and SAPS II (per-point, OR = 1.08, 95%CI = 1.05–1.11, p<0.001) and negatively with biliary infections (OR = 0.06, 95%CI = 0.01–0.48,p = 0.008). In this study, the antimicrobial susceptibility pattern of bacteria isolated from patients with healthcare contact was shifted to resistance, suggesting the need for consideration of the healthcare category (not including hospital-acquired infections) for severe IAIs. 30-day mortality was positively related with age >75 years, severity and *Candida* isolation but not with AMR.

## Introduction

Complicated intra-abdominal infections (IAIs) are severe infections requiring early adequate empirical treatment in addition to control of infectious foci. Approximately 40% of all patients diagnosed with secondary peritonitis need ICU treatment [1], IAIs being the second most common cause of sepsis [2]. Reported mortality rates of severe secondary peritonitis have only slightly decreased over the last decades, and range from 20% to 60% [1,3], thus remaining as an important field for research. The increasingly advanced age of the population (with the associated burden of chronic diseases), and the increased diagnosis in patients with impaired host defences complicate even more outcomes and increase mortality [3,4].

Since there is general agreement on the prime importance of controlling early source to reduce mortality [4–6], nowadays, the focus is on improving early empirical antimicrobial treatments. Inadequate empirical treatments are those providing inadequate coverage of the potential microorganisms involved, their resistance traits being of special importance. For complicated IAIs, the traditional binary classification (in-/out-hospital patient) may be not fully resolute for identification of patients at risk for antibiotic-resistant infections since non-hospitalized patients having contact with healthcare settings may be nonetheless at risk. Furthermore, resistant isolates are increasingly being isolated from patients without risk factors for healthcare/hospital- acquired infections [4,7,8], this representing a challenge for selecting initial antibiotic treatment.

The AGORA initiative [9] and the 2017’s guidelines by The Surgical Infection Society [4] differentiate community-acquired IAIs and healthcare-associated IAIS, with presumed differences in resistance patterns between both categories [9]. But in both documents, hospital-acquired infections are considered as part of healthcare-associated infections [4,9]. In IAIs the concept of healthcare-associated infections as opposed to hospital-acquired infections (unlike what occurs for bacteraemia and pneumonia) is scarcely supported by published data [10] and, traditionally, it is not considered a category different from nosocomial and community-acquired IAIs [11]. However, in contrast to the widely described resistance in pathogens responsible of nosocomial IAIs [12,13], there are few data from healthcare-associated and community-acquired IAIs to help in identifying risks factors for infections by resistant pathogens [4]. In addition, if the presence of multi-drug resistant bacteria implies higher severity, as it occurs for other pathologies [14], needs also further clarification.

We designed the prospective observational multicentre HELP study to describe characteristics of IAIs having community-onset (i.e, non-postoperative/non-nosocomial), in patients that due to their severity required ICU management, to investigate possible differences in antibiotic resistance patterns between patients with and without healthcare exposure, and factors associated with mortality.

## Material and methods

The HELP study (HELP = HEaLthcare-associated intra-abdominal infections study in critically ill Patients) was a prospective, observational, multicentre study conducted in 17 ICUs in Spanish Universitary hospitals. These centres included all consecutive adult patients (≥18 yr), admitted in the ICU after surgical treatment for control of the infectious foci, having as diagnosis non-postoperative/non-nosocomial IAIs. All patients must have an intraoperative microbiological sample (peritoneal fluid) collected for culture. The study included patients entering the ICUs between November 1^st^ 2014 and October 31^th^ 2015. IAIs following surgery (post-operative) or those diagnosed in patients admitted for ≥48 hours (nosocomial) were excluded. The study protocol was approved by the Ethics Committee of Hospital La Paz, Madrid, Spain. Participants were asked to sign a written consent at entry in the ICU. The study was performed in accordance with the ethical standards laid down in the Declaration of Helsinki (Edinburgh, October 2000).

Patients were divided in three groups: patients with healthcare-associated infections (HCAI), patients with community-acquired infections (CAI) and immunocompromised patients (ICP). HCAI was defined as an infection in patients with at least one of the following criteria: i) Hospitalization in an acute care hospital for ≥48 hours within 90 days prior to the onset of infection, ii) Residence in nursing homes or extended-care facilities, iii) Home infusion therapy including antibiotics, iv) Chronic dialysis within 30 days prior to the onset of infection, v) Domiciliary wound care, and/or vi) Family member carrying multidrug-resistant pathogens [15]. ICP were considered patients with immunosuppressive treatment, chemotherapy, corticosteroid therapy for at least 4 weeks before onset of infection, solid organ transplant, and/or HIV-positive patients [15]. IAIs that did not fill exclusion criteria and criteria for HCAI or ICP were considered CAI. Patients presenting criteria for classification as ICP and HCAI were assigned to the HCAI group.

Demographic, clinical and microbiological data, empirical antimicrobial treatment, complications, length of stay, and outcome (mortality in the ICU and 30-day mortality) were recorded. Adequacy of source control was evaluated considering criteria previously described [16]. The previous functional status (Barthel index) [17], the Sequential Organ Failure Assessment (SOFA) [18] and the Simplified Acute Physiologic Score-II (SAPS II) [19] scores were calculated using clinical data at ICU admission. Blood and intraoperative intra-abdominal fluid samples were collected and immediately sent to the Microbiology laboratory of each centre for processing following standard methods in daily practice. Isolated bacteria presenting extended-spectrum β-lactamases (ESBL) and/or carbapenemase production (for gram-negatives) and/or high-level aminoglycoside resistance (HLAR) and/or resistance to vancomycin and/or methicillin resistance (for gram-positives) were considered antimicrobial resistant pathogens (AMR).

Differences between groups were assessed by t-test or U-Mann-Whitney non-parametric tests (continuous variables) or by Chi square/Fisher exact tests (discrete variables). For the comparisons between the three groups (HCAI, CAI and ICP), due to the multiple comparisons performed, the Bonferroni correction was applied and the resulting level of significance was rounded down and set at p ≤ 0.01. A stepwise logistic regression multivariate analysis was conducted in order to determine variables associated with 30-day mortality. All variables showing differences in the two-group data (survivors/dead) analyse (p <0.05) were included. Interactions and linear dependence between independent variables were previously controlled. The significant model (p <0.05) showing the maximum parsimony (the lowest number of variables without significant reduction in the value of the determination coefficient) and the highest R^2^ was considered. All statistical calculations were computed using SPSS v.14.

## Results

A total of 360 outpatients with IAIs (non-postoperative/non-nosocomial) were considered in the 17 participating ICUs during the one-year study period. Fifteen were excluded due to missing data, and 345 patients remained as evaluable for the study analysis. Of them, 18.8% (65 out of 345) were HCAI cases, 6.1% (21 out of 345) were ICP cases, and the remaining 75.1% (259 out of 345) were cases of CAI. Up to 51.6% patients presented generalized peritonitis, without difference by groups. Table 1 shows demographic data by type of infection. Source control was considered adequate in 311 (90.1%) cases, without differences by study group. Colon was the most frequent infection site in all groups: up to ~62% cases among ICPs, a percentage higher than for CAI (32.8%) (p = 0.007). Patients with HCAI presented significantly higher mean age (p<0.001) and abscess formation (p = 0.004) while patients with CAI presented higher percentage of infections involving the appendix compared to HCAI (p<0.001).

**Table 1:** Demographic and clinical data.

Variable	HCAI^a^ (n = 65)	CAI^a^ (n = 259)		ICP^a^ (n = 21)		
			p vs. HCAI		p vs. HCAI	p vs. CAI
**Age (years; mean ± SD)**	72.5 ± 15.2	62.3 ± 17.5	<0.001	61.0 ± 16.2	ns	ns
**Age >75 years [n (%)]**	36 (55.4)	72 (27.8)	<0.001	4 (19.0)	0.003	ns
**Males**	35 (53.8)	143 (55.2)	ns	13 (61.9)	ns	ns
**Days in hospital pre-ICU admission (mean ± SD)**	2.3 ± 8.4	0.6 ± 0.9	ns	1.0 ± 1.5	ns	ns
**Adequate control of infectious foci**	58 (89.2)	236 (91.1)	ns	17 (81.0)	ns	ns
**Site of infection**						
Stomach	9 (13.8)	47 (18.1)	ns	0 (0.0)	ns	ns
Small bowel	13 (20.0)	28 (10.8)	ns	2 (9.5)	ns	ns
Biliary tract	18 (27.7)	43 (16.6)	ns	3 (14.3)	ns	ns
Appendix	2 (3.1)	56 (21.6)	<0.001	3 (14.3)	ns	ns
Colon	23 (35.4)	85 (32.8)	ns	13 (61.9)	ns	0.007
**Type of infection/process**						
Generalized peritonitis	32 (49.2)	131 (50.6)	ns	15 (71.4)	ns	ns
Localized peritonitis	15 (23.1)	83 (32.0)	ns	3 (14.3)	ns	ns
Abscess	12 (18.5)	18 (6.9)	0.004	0 (0.0)	ns	ns
GI^b^ perforation	6 (9.2)	27 (10.4)	ns	3 (14.3)	ns	ns
**Negative peritoneal culture**	7 (10.5)	48 (18.5)	ns	0 (0.0)	ns	ns

Demographic and clinical data by type of peritonitis. Data expressed as n (%) except where indicated

^**a**^HCAI: Healthcare-associated infection; CAI: Community-associated infection; ICP: Immunocompromised patients

^**b**^GI: Gastro-intestinal

ns: non-significant (p>0.01)

Table 2 shows comorbidities, severity scores and complications by type of infection. Significantly higher percentages of patients with congestive heart disease, chronic renal failure, and malignancies were found in the HCAI versus CAI groups; values of SAPS II and SOFA were also higher (p<0.001). Malignancies and liver disease were more frequent among ICP versus CAI cases (p<0.001). Septic shock was the most frequent complication in all groups, being significantly more frequent in HCAI (>50% cases) than in CAI.

**Table 2:** Comorbidities, severity scores and complications.

Variable	HCAI^a^ (n = 65)	CAI^a^ (n = 259)		ICP^a^ (n = 21)		
			p vs. HCAI		p vs. HCAI	p vs. CAI
**Barthel index (mean ± SD)**	71.8 ± 24.9	89.3 ± 13.1	<0.001	85.2 ± 12.2	0.007	ns
**Comorbidities**						
Congestive heart disease	12 (18.5)	10 (3.9)	<0.001	3 (14.3)	ns	ns
Chronic Obstructive Pulmonary Disease	8 (12.3)	31 (12.0)	ns	0 (0.0)	ns	ns
Diabetes mellitus	13 (20.0)	42 (16.2)	ns	3 (14.3)	ns	ns
Liver disease	3 (4.6)	12 (3.6)	ns	6 (28.6)	0.002	<0.001
Chronic renal failure	12 (18.5)	16 (6.2)	0.001	3 (14.3)	ns	ns
Alcoholism	11 (16.9)	26 (10.0)	ns	3 (14.3)	ns	ns
Malignancies	17 (26.2)	30 (11.6)	0.003	9 (42.9)	ns	<0.001
**Severity (mean ± SD)**						
SAPS II^b^	46.6 ± 14.7	37.0 ± 16.0	<0.001	42.3 ± 17.4	ns	ns
Sequential Organ Failure Assessment (SOFA)	6.3 ± 3.1	4.2 ± 3.6	<0.001	6.3 ± 2.5	ns	ns
Lactate (mmol/l)	2.5 ± 2.8	2.4 ± 2.2	ns	2.4 ± 2.2	ns	ns
**Complications**						
Acute respiratory distress syndrome	4 (6.2)	18 (6.9)	ns	0 (0.0)	ns	ns
Mechanical ventilation >24h	20 (30.8)	54 (20.8)	ns	8 (38.1)	ns	ns
Noninvasive mechanical ventilation>24h	7 (10.8)	29 (11.2)	ns	1 (4.8)	ns	ns
Septic shock	33 (50.8)	79 (30.5)	0.002	12 (57.1)	ns	ns
Disseminated intravascular coagulation	4 (6.2)	11 (4.2)	ns	0 (0.0)	ns	ns
Acute renal failure	24 (36.9)	70 (27.0)	ns	9 (42.9)	ns	ns
Renal replacement therapy	4 (6.2)	19 (7.3)	ns	3 (14.3)	ns	ns

Comorbidities, severity scores and complications. Data expressed as n (%) except where indicated

^**a**^HCAI: Healthcare-associated infection; CAI: Community-associated infection; ICP: Immunocompromised patients

^**b**^SAPS II: Simplified Acute Physiologic Score-II

ns: non-significant (p>0.01)

Only 10.7% patients presented positive blood culture; among them, the most frequent isolate was *E*. *coli* (3.2%), without differences between groups. Among bacteria isolated from peritoneal fluid, the percentage of AMR was 11.0%, being 7.0% for ESBL- and/or carbapenemase- producing enterobacteria. Overall, the percentage of AMR was significantly higher in HCAI (23/65; 35.4%) vs. CAI (15/259; 5.8%) (p<0.001) or vs. ICP (0/21; 0%) (p = 0.003), without differences between CAI and ICP (p = 0.641).

Table 3 shows per species percentage of isolation of aerobic/facultative bacteria from peritoneal fluid. Isolation of ESBL-and/or carbapenemase- producing bacteria was significantly more frequent from HCAI than CAI patients (p<0.001), with significant higher percentages of ESBL-producing *E*. *coli* (p = 0.030) and *Klebsiella* spp. (p = 0.001). These type of isolates were not found among enterobacteria isolated from ICP patients. Isolation of enterococci was significantly more frequent from HCAI than CAI (33.8% vs. 17.8%, p = 0.004), with higher percentage of isolates showing HLAR [9 out of 22 (40.9%) vs. 3 out of 46 (6.5%) for HCAI vs. CAI isolates].

**Table 3:** Microbiological data.

Bacteria	HCAI^a^ (n = 65)	CAI^a^ (n = 259)		ICP^a^ (n = 21)		
			p vs. HCAI		p vs. HCAI	p vs. CAI
**Total Enterobacteria**	48 (73.8)	169 (65.3)	ns	17 (81.0)	ns	ns
***E*. *coli* (Total)**	31 (47.7)	119 (45.9)	ns	13 (61.9)	ns	ns
*E*. *coli* (ESBL^b^)	5 (7.7)	5 (1.9)	ns	0 (0.0)	0.009	ns
*E*. *coli* (CPE^c^)	2 (3.1)	2 (0.8)	ns	0 (0.0)	ns	ns
***Klebsiella* spp (Total)**	11 (16.9)	33 (12.7)	ns	3 (14.3)	ns	ns
*Klebsiella* spp (ESBL^b^)	4 (6.2)	0 (0.0)	0.001	0 (0.0)	ns	ns
*Klebsiella* spp (CPE^c^)	1 (1.5)	0 (0.0)	ns	0 (0.0)	ns	ns
***Enterobacter* spp (Total)**	3 (4.6)	11 (4.2)	ns	1 (4.8)	ns	ns
*Enterobacter* spp (ESBL^b^)	1 (1.5)	4 (1.5)	ns	0 (0.0)	ns	ns
**Total ESBL**^b^ **enterobacteria**	10 (15.4)	9 (3.5)	0.001	0 (0.0)	ns	ns
**Total CPE**^c^	3 (4.6)	2 (0.8)	ns	0 (0.0)	ns	ns
**Total ESBL**^b^ **and/or CPE**^c^	13 (20.0)	11 (4.2)	<0.001	0 (0.0)	ns	ns
***Pseudomonas* spp.**	1 (1.5)	16 (6.2)	ns	3 (14.3)	ns	ns
**Total gram-positive bacteria**	39 (60.0)	112 (43.2)	ns	7 (33.3)	ns	ns
**Total *Enterococcus***	22 (33.8)	46 (17.8)	0.004	2 (9.5)	ns	ns
*E*. *faecalis*	5 (7.7)	17 (6.6)	ns	1 (4.8)	ns	ns
*E*. *faecium* (Total)	11 (16.9)	16 (6.2)	0.005	1 (4.8)	ns	ns
*E*. *faecium* (HLAR^d^)	6 (9.2)	1 (0.4)	<0.001	0 (0.0)	ns	ns
*Enterococcus* spp. (Total)	6 (9.2)	13 (5.0)	ns	0 (0.0)	ns	ns
*Enterococcus* spp. (HLAR^d^)	3 (4.6)	2 (0.8)	ns	0 (0.0)	ns	ns
*Enterococcus* spp. (vancomycin-resistant)	1 (1.5)	1 (0.4)	ns	0 (0.0)	ns	ns
**Total HLAR**^d^ **bacteria**	9 (13.8)	3 (1.2)	<0.001	0 (0.0)	ns	ns

Per species, percentage of isolation of aerobe/facultative bacteria from peritoneal fluid. Data are expressed as n (%).

^**a**^HCAI: Healthcare-associated infection; CAI: Community-associated infection; ICP: Immunocompromised patients

^**b**^ESBL: extended spectrum β-lactamase-producing

^**c**^CPE: carbapenemase-producing

^**d**^HLAR: High-level aminoglycoside resistance

ns: non-significant ns: non-significant (p>0.01)

No differences in total gram-negative anaerobic bacteria isolation were found between groups. ICP patients presented significantly higher rates of *Clostridium* spp. isolation compared with patients with CAI (23.8% vs. 4.7%; p = 0.005). Overall, isolation of *Candida* spp. occurred in 19.1% (66 out of 345) patients, without differences between groups (23.1% for HCAI, 17.8% for CAI and 23.8% for ICP).

Table 4 shows empirical antimicrobial treatment by group. Piperacillin-tazobactam was significantly more frequent as empirical treatment in CAI than in ICP (p = 0.008), while the use of carbapenems (and among them, meropenem) was significantly more frequent in ICPs vs. HCAI or CAI (p<0.01). Treatment with tigecycline and daptomycin was significantly more frequent in HCAI and ICP than in CAI patients. Antifungals were used in a total of 13% patients, without differences between groups.

**Table 4:** Antimicrobial treatment.

Antimicrobials	HCAI^a^ (n = 65)	CAI^a^ (n = 259)		ICP^a^ (n = 21)		
			p vs. HCAI		p vs. HCAI	p vs. CAI
**Total β-lactams**	62 (0.95)	266 (1.03)	ns	22 (1.05)	ns	ns
** Penicillins (Total)**	33 (0.51)	165 (0.64)	ns	6 (0.29)	ns	0.001
Amoxicillin/clavulanic acid	5 (0.08)	56 (0.22)	ns	0 (0.00)	ns	ns
Piperacillin/tazobactam	28 (0.43)	109 (0.42)	ns	6 (0.29)	ns	0.008
** Cephalosporins (Total)**	4 (0.06)	21 (0.08)	ns	2 (0.10)	ns	ns
Cefotaxime	3 (0.05)	18 (0.07)	ns	1 (0.05)	ns	ns
Ceftriaxone	1 (0.02)	2 (0.01)	ns	1 (0.05)	ns	ns
Ceftazidime	0 (0.00)	1 (0.00)	ns	0 (0.00)	-	ns
** Carbapenems (Total)**	23 (0.35)	79 (0.31)	ns	14 (0.67)	ns	0.001
Imipenem	1 (0.02)	15 (0.06)	ns	1 (0.05)	ns	ns
Meropenem	17 (0.26)	35 (0.14)	ns	12 (0.57)	0.009	<0.001
Ertapenem	5 (0.08)	29 (0.11)	ns	1 (0.05)	ns	ns
** Monobactams (Aztreonam)**	2 (0.03)	1 (0.00)	ns	0 (0.00)	ns	ns
**Total aminoglycosides**	4 (0.06)	15 (0.06)	ns	2 (0.10)	ns	ns
**Total quinolones**	2 (0.03)	5 (0.02)	ns	1 (0.05)	ns	ns
**Glycylcyclines (Tigecycline)**	9 (0.14)	7 (0.03)	<0.001	3 (0.14)	ns	0.006
**Daptomycin**	4 (0.06)	2 (0.01)	0.004	2 (0.10)	ns	0.001
**Linezolid**	4 (0.06)	16 (0.06)	ns	4 (0.19)	ns	ns
**Metronidazole**	7 (0.11)	27 (0.10)	ns	3 (0.14)	ns	ns
**Total antifungals**	10 (0.15)	29 (0.11)	ns	6 (0.29)	ns	ns

Antimicrobials used as empirical treatment. Data are expressed as n (ratio of per-patient antimicrobial use).

^**a**^HCAI: Healthcare-associated infection; CAI: Community-associated infection; ICP: Immunocompromised patients

ns: non-significant (p>0.01)

Table 5 shows length of stay and outcome by type of infection. No differences in the variables analysed were found between groups. In the subgroup of patients >75 years (n = 112), no significant differences (p = 0.452) in 30-day mortality were found between groups: 12/36 (33.3%) for HCAI; 19/72 (26.4%) for CAI and 0/4 for ICP.

**Table 5:** Length of stay and outcome in the ICU.

Variable	HCAI^a^ (n = 65)	CAI^a^ (n = 259)		ICP^a^ (n = 21)		
			p vs. HCAI		p vs. HCAI	p vs. CAI
**Length of stay in the ICU [days; median (IQR)]**	4.0 (1.0–7.0)	2.0 (1.0–6.0)	ns	5.0 (1.0–8.5)	ns	ns
**Length of hospital stay [days; median (IQR)]**	17.0 (10.5–27.0)	12.0 (8.0–24.0)	ns	19.0 (13.0–46.5)	ns	ns
**Mortality in the ICU [n (%)]**	3 (4.6)	21 (8.1)	ns	4 (19.0)	ns	ns
**Mortality within 30 days of infection onset [n (%)]**	15 (23.1)	30 (11.6)	ns	5 (23.8)	ns	ns

Length of stay and outcome by type of peritonitis

^**a**^HCAI: Healthcare-associated infection; CAI: Community-associated infection; ICP: Immunocompromised patients

IQR: Interquartile range

ns: non-significant (p>0.01)

The ICP group was excluded from the multivariate analysis due to its low number of patients, remaining 324 patients (279 surviving and 45 dead patients). Variables showing differences in the two-group data analysis (those included in the multivariate analysis) are shown in Table 6. Different models were performed. The Hosmer-Lemeshow test and the performance of the models were evaluated, being always <0.01. The significant (p<0.05) model showing the maximum parsimony and the highest R^2^ (R^2^ = 0.262, p = 0.021) indicated age >75 years (OR = 6.67, 95%CI = 2.56–17.36, p<0.001), *Candida* isolation from peritoneal fluid (OR = 3.05, 95%CI = 1.18–7.87, p = 0.022), and SAPS II (per point, OR = 1.08, 95%CI = 1.05–1.11, p<0.001) as variables positively associated with mortality, and biliary tract as site of infection (OR = 0.06, 95%CI = 0.01–0.48, p = 0.008) as variable negatively associated with mortality.

**Table 6:** Significant data in two-group data analysis.

Variable	Survivors (n = 279)	Dead (n = 45)	p
**Age >75 years**	77 (27.6)	31 (68.9)	<0.001
**HCAI**^a^	50 (17.9)	15 (33.3)	0.017
**Small bowel as site of infection**	29 (10.4)	12 (26.7)	0.002
**Biliary tract as site of infection**	60 (21.5)	1 (2.2)	0.002
**Appendix as site of infection**	55 (19.7)	3 (6.7)	0.034
**Generalized peritonitis**	132 (47.3)	31 (68.9)	0.007
**Localized peritonitis**	93 (33.3)	5 (11.1)	0.003
**Congestive heart disease**	14 (5.0)	8 (17.8)	0.005
**Chronic renal failure**	18 (6.5)	10 (22.2)	0.002
**Lactate ≥3 mmol/l**	59 (21.1)	20 (44.4)	0.001
**SAPS II**^b^ **>46 points**	63 (22.6)	32 (71.1)	<0.001
**Isolation of CPE**^c^ **from peritoneal fluid**	2 (0.7)	3 (6.7)	0.021
**Isolation of *Enterobacter* spp. from peritoneal fluid**	5 (1.8)	4 (8.9)	0.024
**Isolation of *Candida* spp. from peritoneal fluid**	40 (14.3)	13 (28.9)	0.027

Variables (values at admission) introduced in the multivariate model for the HCAI^a^ + CAI^a^ population based on the significant (p<0.05) differences between survivors and dead patients. Data expressed as n (%).

^**a**^HCAI: Healthcare-associated infection; CAI: Community-associated infection

^**b**^SAPS II: Simplified Acute Physiologic Score-II

^**c**^CPE: carbapenemase-producing Enterobacteria

## Discussion

Up to 17 Spanish UCIs participated in this observational study analysing severe IAIs having community-onset. Our results confirm for this entity the difference of resistance patterns in community patients with and without healthcare exposure, since isolation of AMR from peritoneal fluid was significantly more frequent from HCAI versus CAI. However, in the multivariate analysis no association between AMR and 30-day mortality was found. On the contrary, our 30-day mortality was positively associated with age >75 years, *Candida* isolation from peritoneal fluid, and values of SAPS II, and negatively associated with biliary tract as site of infection.

Therapeutic recommendations are based, among others, on the severity of the infection, age, patient’s status captured by scoring systems [4,20], and the possibility of multidrug resistant bacteria in relation to the origin of the infection [4,21]. In the present study, the different characteristics of patients in the study groups were in line with all these criteria: patients with healthcare exposure were significantly older, presented higher severity at admission, and presented more frequently AMR than CAI cases. Therefore, from the microbiological perspective (AMR), our results validate the category of healthcare-associated IAIs as opposed to community infections. This constitutes the main difference with other studies investigating risk factors for IAIs by multidrug resistant pathogens which considered hospital-acquired infections among healthcare-associated infections [22,23].

The profile of pathogens isolated from peritoneal samples was in accordance with previous studies, with similar percentages of enzymatic resistance in enterobacteria (20% for HCAI and 4.2% for CAI) [24,25], and isolation rates of enterococci significantly higher in HCAI than CAI, as in a previous study on community-onset healthcare-associated IAIs [26]. An interesting finding was the low rate of non-fermenters found, probably in relation to the community-onset of infections, since additional pathogenic bacteria as *P*. *aeruginosa* and yeasts must be considered in hospital-associated complicated IAIs and in immunocompromised hosts [27]. The reduced number of ICP patients limited its analysis, not allowing for drawing conclusions. *Candida* spp. was the third most frequent microorganism isolated after *E*. *coli* and enterococci, without differences in isolation rates between HCAI and CAI. The overall percentage of *Candida* isolation in our study was high, and similar to the percentage found in critically ill patients with secondary and tertiary abdominal sepsis [28]. Nevertheless, postoperative peritonitis has different microbiological features than non-postoperative nosocomial IAIs and CAIs, where the similar frequency of yeast isolation has been reported [29].

Surprisingly, AMR (as defined in the present study) were not isolated from ICP patients, possibly due to the reduced number of ICP cases included. But, on the contrary, AMR were found in 5.8% of the patients not having contact with healthcare, thus, theoretically, not presenting risk factors for harbouring resistant bacteria. In this sense, the lack of difference in isolation of ESBL-producing *E*. *coli* between HCAI and CAI illustrates the extension of the risk for resistance beyond the healthcare settings to the community. The presence of resistant *E*. *coli* isolates in the community is increasing over time, as confirmed in the SMART Program, where the rate of multidrug-resistant *E*. *coli* was more than double from 2009 to 2013 among community-associated IAIs [30].

In the present study, overall mortality was similar to the one described in other studies on non-post-operative intraabdominal infections [31,32]. The multivariate analysis did not identify AMR as variable associated with 30-day mortality. A previous study in ICU-acquired bloodstream infections showed similar results, the pathogen resistance pattern not impacting attributable mortality [33]. In our study, the 30-day mortality relied on other classical parameters as advanced age and severity, and microbiological parameters as isolation of *Candida* spp. Nevertheless, despite its statistical significance, interpretations of these results need caution since for age a substantial OR value was accompanied by a large confidence interval, and for SAPS II the magnitude of effect was an OR = 1.08 per point increased in the score. However, our results are well supported by the literature. In a previous study on acute secondary peritonitis, age older than 80 years showed to be an independent risk factor of mortality regardless the Mannheim Peritonitis Index score [34]. Similarly, several studies have concluded the increased mortality associated with isolation of *Candida* [35,36], also in patients with complicated non-postoperative IAIs [32].

The strength of the present prospective descriptive study was the inclusion of all patients with community-onset severe IAIs admitted in a large number of ICUs in Spain over one year. Time of source control was not available in our study, this representing a study limitation. Other inherent limitations of descriptive studies (absence of sample size calculation, variability in routine laboratory procedures, lack of standardized criteria for hospital discharge…) weakness conclusions, although, on the other hand, discloses actual bacterial isolation and therapeutic management of this entity in clinical practice. Importantly, the analysis of the empirical treatments selected by treating physicians suggests an influence of the awareness of possible resistance in certain patients, since there was a more frequent utilization of broad-spectrum antibiotics in the HCAI and ICP groups. This could have negatively influenced the multivariate analysis for AMR and 30-day mortality. In this context, the lack of assessment of the adequacy of empirical antimicrobial treatments represents a study limitation, although without influence on epidemiological AMR results.

## Conclusion

Despite AMR (as defined in the present study) were not isolated from ICP patients (possibly due to the reduced number of ICP cases included), the results of this study showed that the antimicrobial susceptibility pattern of bacteria isolated from patients with healthcare contact was shifted to resistance, suggesting the need for consideration of the healthcare category (not including hospital-acquired infections) for severe IAIs. According to our results, treating physicians facing the management of severe IAIs should consider not only the patient’s age and severity at admission (risk factors for mortality) but also healthcare exposure as risk factor for multidrug resistance even in IAIs having community-onset.
